# Blocking triggering receptor expressed on myeloid cells-1 attenuates lipopolysaccharide-induced acute lung injury *via* inhibiting NLRP3 inflammasome activation

**DOI:** 10.1038/srep39473

**Published:** 2016-12-22

**Authors:** Tian Liu, Yong Zhou, Ping Li, Jia-Xi Duan, Yong-Ping Liu, Guo-Ying Sun, Li Wan, Liang Dong, Xiang Fang, Jian-Xin Jiang, Cha-Xiang Guan

**Affiliations:** 1 Department of Physiology, Xiangya School of Medicine, Central South University, Changsha, Hunan 410078, China; 2 Department of Anesthesiology, Affiliated Hospital of Zunyi Medical College, Zunyi, Guizhou 56300, China; 3 Department of Neurology, University of Texas Medical Branch, Galveston, TX 77555, USA; 4 State Key Laboratory of Trauma, Burns, and Combined Injury, Research Institute of Surgery, Daping Hospital, Third Military Medical University, Chongqing, 400042, China

## Abstract

Acute lung injury (ALI) is associated with high mortality and uncontrolled inflammation plays a critical role in ALI. TREM-1 is an amplifier of inflammatory response, and is involved in the pathogenesis of many infectious diseases. NLRP3 inflammasome is a member of NLRs family that contributes to ALI. However, the effect of TREM-1 on NLRP3 inflammasome and ALI is still unknown. This study aimed to determine the effect of TREM-1 modulation on LPS-induced ALI and activation of the NLRP3 inflammasome. We showed that LR12, a TREM-1 antagonist peptide, significantly improved survival of mice after lethal doses of LPS. LR12 also attenuated inflammation and lung tissue damage by reducing histopathologic changes, infiltration of the macrophage and neutrophil into the lung, and production of the pro-inflammatory cytokine, and oxidative stress. LR12 decreased expression of the NLRP3, pro-caspase-1 and pro-IL-1β, and inhibited priming of the NLRP3 inflammasome by inhibiting NF-κB. LR12 also reduced the expression of NLRP3 and caspase-1 p10 protein, and secretion of the IL-1β, inhibited activation of the NLRP3 inflammasome by decreasing ROS. For the first time, these data show that TREM-1 aggravates inflammation in ALI by activating NLRP3 inflammasome, and blocking TREM-1 may be a potential therapeutic approach for ALI.

Acute lung injury (ALI) including acute respiratory distress syndrome (ARDS) is the leading cause of acute respiratory failure and often associated with multiple organ failure^1^. ALI is characterized by an increased permeability of the alveolar-capillary barrier, resulting in lung edema with protein-rich fluid and consequently, poor arterial oxygenation^2^. Despite considerable progress has been made in the therapy of ALI, the mortality rate associated with ALI remains very high^3^. Dysregulation of inflammation driven by excessive innate immune response is recognized as the key process in ALI^4^. Innate immune cells in the lung can recognize and bind to invading pathogens through germline-encoded pattern recognition receptors (PRRs), such as Toll-like receptors (TLRs) and Nod-like receptors (NLRs), elicit an innate immune response and initiate adaptive immunity for the control or elimination of infection through both extracellular and intracellular activation cascades. However, when innate immune response is over-activated, the production of numerous pro-inflammatory cytokines and inflammatory bioactive substances would aggravate lung alveolar epithelial cell injury by disrupting permeability of alveolar-capillary barrier^2^. Thus, PPRs signals need to be precisely regulated to avoid tissue damage.

The NLRs family, pyrin domain containing 3 (NLRP3) inflammasome, is comprised of NLRP3, the adaptor protein apoptosis associated speck like protein (ASC) and pro-caspase-1. NLRP3 inflammasome is a major intracellular multi-protein complex of the innate immune system, and is abundant in lung tissue^5^. Upon activation, NLRP3 inflammasome activates caspase-1, which processes precursor form of cytokines (pro-IL-1β and pro-IL-18) to their mature biologically active and secreted forms (IL-1β and IL-18). These bioactive cytokines play a pivotal role in initiation and amplification of the inflammatory processes of ALI. Antibody neutralization of IL-1β or IL-18 attenuates ALI severity in several different rodent models^6^,^7^. In addition, NLRP3 inflammasome activation is involved in ALI induced by lipopolysaccharide (LPS), hyperoxia or burn^8^,^9^,^10^. Thus, the activation of NLRP3 inflammasome is altered and should be tightly controlled in ALI.

Triggering receptors expressed on myeloid cell-1 (TREM-1) is a member of the immunoglobulin superfamily receptor expressed on myeloid cells, including neutrophils and monocytes. TREM-1 activation can amplify TLRs and NLRs signaling to promote the production of pro-inflammatory cytokines, degranulation of neutrophils, and phagocytosis 11,12,13. Depletion or blocking TREM-1 has shown protective effects in sepsis, ischemia-reperfusion, pancreatitis, inflammatory bowel diseases, Fungal Keratitis and arthritis^14^,^15^,^16^,^17^,^18^,^19^,^20^. Our previous study found that the expression of TREM-1 in LPS-induced ALI mice lung and macrophages are significantly increased, suggesting an important role of TREM-1 in ALI^21^,^22^. Although the pro-inflammatory effect of TREM-1 and its implication in the pathogenesis of inflammatory diseases are emerging, the mechanisms are still poorly understood. Previous study showed that TREM-1 activation can increase LPS-induced IL-1β production in human monocytes^23^, suggesting a regulatory role of TREM-1 in activation of the NLRP3 inflammasome. However, its mechanistic insight remains to be further investigated. Although the natural TREM-1 ligand remains unknown, another member of the TREM-1 family, TLT-1, is found to be able to bind TREM-1, therefore dampening TREM-1 engagement^24^. Studies show that the synthesized TLT-1-derived peptide exhibits anti-inflammatory properties by dampening TREM-1 signaling, and it can be used as a natural TREM-1 inhibitor^25^,^26^,^27^. Therefore, a 12 amino acid antagonistic polypeptide (LR12, LQEEDTGEYGCV) derived from mouse TLT-1 was synthesized to investigate the role of TREM-1 in ALI and NLRP3 activation.

In this study, we presented evidence that blocking TREM-1 by LR12 has protective effects against ALI. LR12 decreased pulmonary inflammation and improved overall survival in LPS-induced ALI mice. In addition, LR12 attenuated activation of the NLRP3 inflammasome. The protective effects by LR12 may be related to inhibition of NF-κB activation and ROS production.

## Materials and Methods

### Mice and experimental protocol

All animal studies were approved by The Ethics Committee of Institute of Clinical Pharmacology at Central South University (Changsha, China) in accordance with the guidelines of National Institutes of Health. All surgeries were performed under anesthesia with an intraperitoneal injection of pentobarbital sodium (50 mg/kg) and necessary efforts were taken to minimize suffering.

For the ALI model, C57BL/6 J mice (Shanghai Laboratory Animal Company, China) were randomly grouped and treated with lipopolysaccharide (LPS) (*E. coli* O111:B4; Sigma; 5 mg/kg) intratracheal injection (*i.t.*) for 6 h. Control animals received saline respectively. For survival study, mice were treated with a lethal dose of LPS (20 mg/kg, *i.t*). Survival rate was monitored every 12 h for 3 days after LPS injection. Mice were received saline, antagonistic TREM-1 peptide LR12 (LQEEDTGEYGCV, 5 mg/kg, SBS Genetech, China) or sequence-scrambled control peptide LRS (YQVGELCTGEED, 5 mg/kg) respectively *via* intravenous injection (*i.v*) 2 h before LPS administration.

### Histological analysis

Lung tissue samples were fixed in 4% paraformaldehyde neutral buffer solution for 24 h, dehydrated in a graded ethanol series, embedded in paraffin, and sliced at 5 μm. The sections were stained with Hematoxylin-Eosin (H&E) and histological scoring was performed blindly for following four parameters: alveolar congestion; hemorrhage; infiltration or aggregation of neutrophils in airspace or vessel wall; and thickness of the alveolar wall/hyaline membrane formation. The severity of Inflammation was graded from 0 to 4^28^.

### Lung wet-to-dry ratio determination

Lung was excised and the blood was removed by blotting the lung with filter papers until dry. After weight the wet tissue, the lung was then placed in an incubator at 60 °C for 24 h to obtain the dry weight. The wet-to-dry ratio of the lung was calculated to assess lung edema.

### Assessment of lung MPO activity

Myeloperoxidase (MPO) activity was measured by MPO assay kit (Nanjing Jiancheng Bio-engineering Institute, China). Briefly, the lung tissues were weighed and cut, followed by homogenizing in phosphate buffer (pH6.0) containing 0.5% hexadecyltrimethyl ammonium hydroxide, and then centrifuged. The supernatants were collected and MPO activity was analyzed according to manufacturer’s instructions. The protein concentration of the lung homogenate supernatants was measured using a Bicinchoninic acid (BCA) Protein Assay Kit (Beyotime Biotechnology, China). MPO activity of the supernatants was expressed as units per gram of total protein (U/g).

### Measurement of superoxide dismutase and malondialdehyde

Lung tissues were homogenized in phosphate buffer at a ratio of 1:10 (weight: volume). The lipid peroxide level and superoxide dismutase (SOD) activity were measured using a commercially available kit following manufacturer’s instructions (Nanjing Jiancheng Bio-engineering Institute, China). The lipid peroxide level was expressed as nmol of malondialdehyde (MDA) per mg of tissue protein, and SOD activity was expressed as unit (U) per mg protein.

### LDH activity analysis and cell count

Mice were sacrificed 6 h after LPS injection. Serum samples were collected. Bronchoalveolar lavage fluid (BALF) was obtained *via* cannulation of the trachea and lavaging the airway lumen with 0.8 mL ice-cold phosphate-buffered saline (PBS) for three times. The recovered fluid was centrifuged and the supernatant was collected. The cell pellets re-suspended in PBS to count the number of total cells, neutrophils, and macrophages. Lactate dehydrogenase (LDH) activity in serum and BALF was determined with LDH Cytotoxicity Assay Kit (Nanjing Jiancheng Bio-engineering Institute, China).

### Cytokines measurement

The levels of IL-1β in the serum and BALF, tumor necrosis factor-α (TNF-α) and IL-10 in the BALF were measured by commercially available ELISA assay kits (Sigma, USA), according to the manufacturer’s instructions.

### Real-time PCR

Total RNA in the lung was extracted using TRIzol (TaKaRa, Japan) and was quantified by spectrophotometric analysis using an ultraviolet spectrophotometer (Thermo Scientific, USA). The generation of cDNA from RNA (2 μg) was performed using RevertAid First t Strand cDNA short Kit (Thermo Scientific, USA) according to the manufacturer’s instructions. Real-Time PCR was performed using UltraSYBR Mixture (TaKaRa, Japan) on Bio-Rad real-time PCR system (CFX96 Touch™, Bio-Rad, USA). Each experiment was performed in triplicate in three replicate wells. The real-time PCR data was analyzed using the comparative CT method (2^−ΔΔCT^). All results were normalized using β-actin as an internal standard.

### Western blotting

Lung tissues were homogenized, separated on 12% SDS-PAGE gel, and then transferred onto a PVDF membrane. The membrane was blocked in 5% fat-free milk and probed with primary antibody against pro-IL-1β (Cell Signaling Technology, USA), IL-1β p17, NLRP3, caspase-1 p10 (Santa Cruz, USA) and IκB (Beyotime Biotechnology, China). Horseradish peroxidase-conjugated secondary antibodies (Santa Cruz, USA; Cell Signaling Technology, USA) and enhanced chemiluminescence were applied to detect protein content. Images were collected using ChemiDoc XRS (Bio-Rad, USA). Bands were quantified using Image J.

### Immunofluorescent assay

Paraffin slides of the lung tissues were deparaffinized, and antigen retrieval was achieved by microwave treatment in 1 mM EDTA (pH 9.0) once at 900 W for 7 min and twice at 440 W for 5 min. After cooling for 20 min at room temperature, the slides were washed with PBS 3 times, 5 min for each time. To avoid nonspecific reactions, slides were treated with 3% bovine serum albumin in PBS/0.3% Triton X-100 for 30 min at room temperature. After washing and draining, the slides were incubated with Anti-NLRP3 antibody (Santa Cruz, USA) for 15 min at room temperature, then overnight at 4 °C. After 3 time washes with PBS, these slides were incubated with Donkey Anti-Goat IgG at a 1:200 dilution in 2% bovine serum albumin for 1 h at 37 °C in the dark. The nucleus was stained with DAPI for 10 min in the dark.

### Statistical analysis

All values are expressed as the mean ± standard deviation. Statistical analyses were performed using one-way analysis of variance (ANOVA) for multiple comparisons. The SNK test served as the post hoc test for multiple comparisons. Survival rate was evaluated by the Kaplan-Meier test. A two-tailed *P* value of less than 0.05 was considered to be statistically significant.

## Results

### LR12 reduced mortality and lung pathological changes in LPS-induced ALI mice

**Figure 1:**
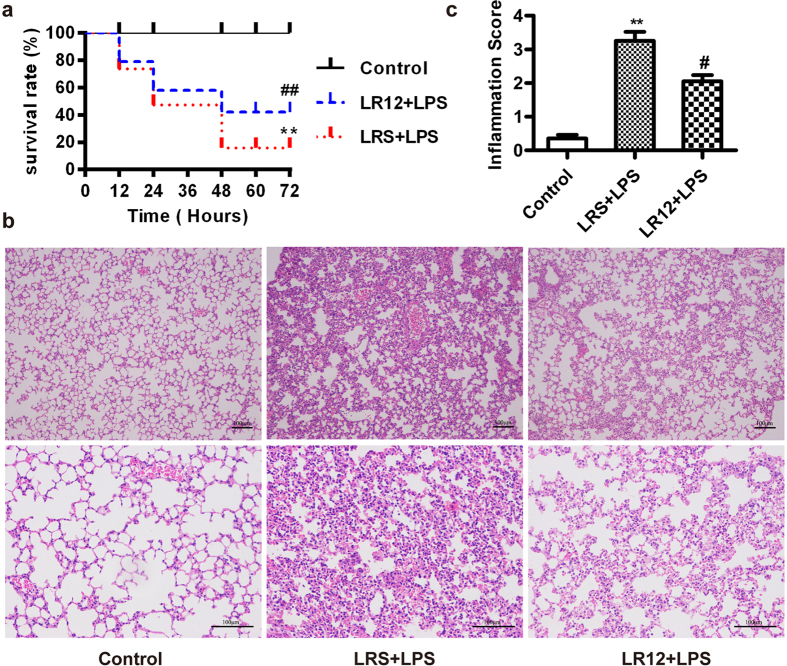
LR12 reduced mortality and lung pathological changes in LPS-induced ALI mice. Mice were treated with saline, LR12 (5 mg/kg) and LRS (5 mg/kg) intravenous injection, respectively. Two hours later, mice were given either saline or a lethal dose of LPS (20 mg/kg, *i.t*.) to monitor survival (n = 20) every 12 h for 3 days (**a**), or a sub-lethal dose of LPS (5 mg/kg*, i.t.*) for 6 h to detect the lung histopathological change by H&E staining (**b**, Bar = 100 μm) and lung inflammation score (**c**, n = 4–7). Data are expressed as the mean ± SEM. **P < 0.01 *vs* Control group, ^#^*P* < 0.05 *vs* LRS+LPS group.

Our preliminary experiments showed that either antagonistic TREM-1 peptide (LR12) or sequence-scrambled control peptide (LRS) alone did not alter morphological characteristics, pro-inflammatory cytokine expression and cellular damage of lung in the mice treated with normal saline (Fig. s1). To determine the protective effects of TREM-1 inhibition on LPS-induced ALI, we investigated whether LR12 (5 mg/kg, *i.v.*) could improve survival in ALI mice induced by a lethal dose of LPS (20 mg/kg, *i.t*). Only 47.4% mice treated with LPS survived at first 24 h, and the survival rate at 48 h was 15.8%, while LR12 improved survival rate to 57.9% at 24 h and 43.4% at 48 h. There were no late deaths after 60 h (Fig. 1a). We then determined the effects of LR12 on the lung pathological changes in a sub-lethal dose of LPS (5 mg/kg, *i.t.*) challenged mice. Mice were sacrificed 6 h after LPS injection and lung tissues were stained with H&E. Lung histology showed that LPS produced remarkable lung inflammatory responses including significant interstitial infiltration of inflammatory cells and thickening of the alveolar walls. The pathological changes of lung were significantly alleviated by LR12 treatment (Fig. 1b,c).

### LR12 reduced lung permeability and damage in mice induced by LPS

**Figure 2:**
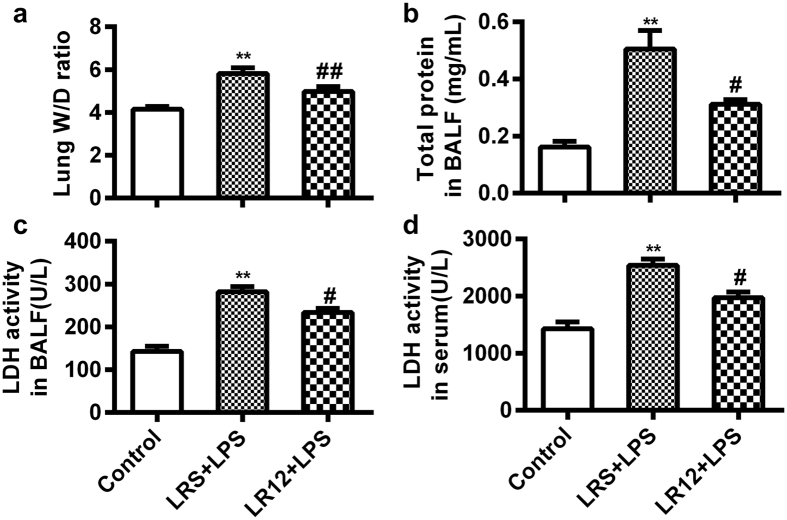
LR12 reduced lung permeability and damage in mice induced by LPS. Mice received saline, LR12 (5 mg/kg) and LRS (5 mg/kg) intravenous injection, respectively. Two hours later, mice were treated with either saline or LPS (5 mg/kg, *i.t*.) for 6 h. Lung W/D weight ratio (**a**), total protein in BALF (**b**) were measured to determine lung permeability. LDH activity in BALF (**c**) and serum (**d**) was determined to assess lung damage. (n = 4–7). Data are expressed as the mean ± SEM. *P < 0.05 and **P < 0.01 vs Control group, ^#^*P* < 0.05 and ^##^*P* < 0.01 vs LRS+LPS group.

Disruption of the alveolar-capillary barrier can increase lung permeability and facilitate protein penetration, resulting in lung edema, eventually acute respiratory failure. We measured the lung wet-to-dry ratio and total protein in BLAF to determine the degree of lung edema and dysregulated barrier function^29^,^30^. The results showed that LPS significantly increased the lung wet-to-dry ratio (Fig. 2a) and the total protein concentration in BALF of mice (Fig. 2b). LR12 remarkably reversed lung edema and total protein concentration induced by LPS. In addition, LDH activity, an index for cellular damage, was increased after LPS treatment, while this effect was remarkably attenuated by LR12 administration (Fig. 2c,d). These data suggest LR12 can improve the integrity of alveolar-capillary barrier impaired by LPS.

### LR12 suppressed accumulation of the inflammatory cells in the lung treated with LPS

**Figure 3:**
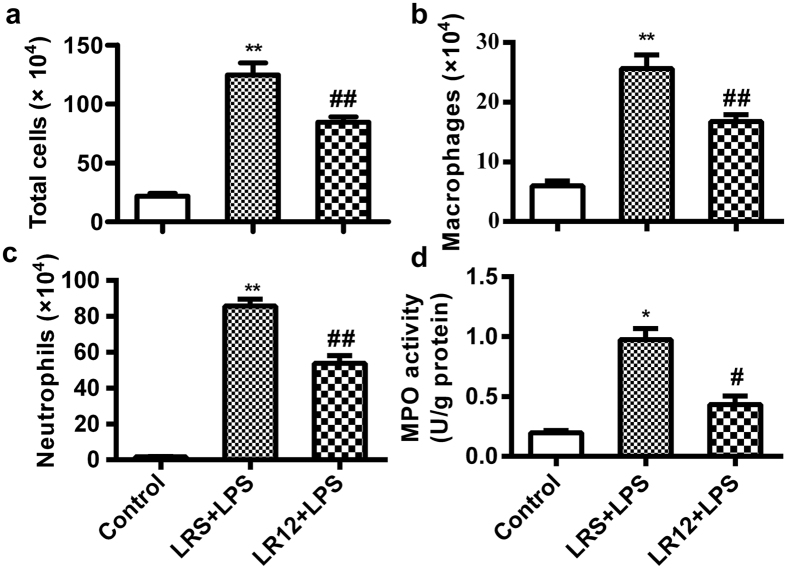
LR12 suppressed accumulation of the inflammatory cells in the lung treated with LPS. Mice received saline, LR12 (5 mg/kg) and LRS (5 mg/kg) intravenous injection, respectively. Two hours later, mice were treated with either saline or LPS (5 mg/kg, *i.t*.) for 6 h. Total cells (**a**), macrophages (**b**), neutrophils (**c**) in BALF and MPO activity (**d**) in lung tissues were determined (n = 4–7). Data are expressed as the mean ± SEM. *P < 0.05 and **P < 0.01 vs Control group, ^#^*P* < 0.05 and ^##^*P* < 0.01 vs LRS+LPS group.

Inflammatory cells, especially neutrophils infiltrated into the lung after disruption of the alveolar-capillary barrier, would be activated, and these cells subsequently release pro-inflammatory cytokines and toxic mediators. These bioactive substances can impair the endothelial-epithelial barriers of the lung^31^. Therefore, we determined the role of LR12 in inflammatory cells infiltration in the lung of LPS-challenged mice. The number of total cells (Fig. 3a), macrophages (Fig. 3b) and neutrophils (Fig. 3c) in BALF were significantly increased after LPS challenge, which were significantly decreased by LR12 administration. MPO activity is an important index of neutrophil migration into the lung^32^. The activity of MPO (Fig. 3d) in the lung tissues significantly increased after LPS administration, which was reduced by LR12. The findings suggest that LR12 suppresses infiltration of the inflammatory cells in LPS-induced ALI.

### LR12 altered TNF-α and IL-10 expression in LPS-induced ALI mice

**Figure 4:**
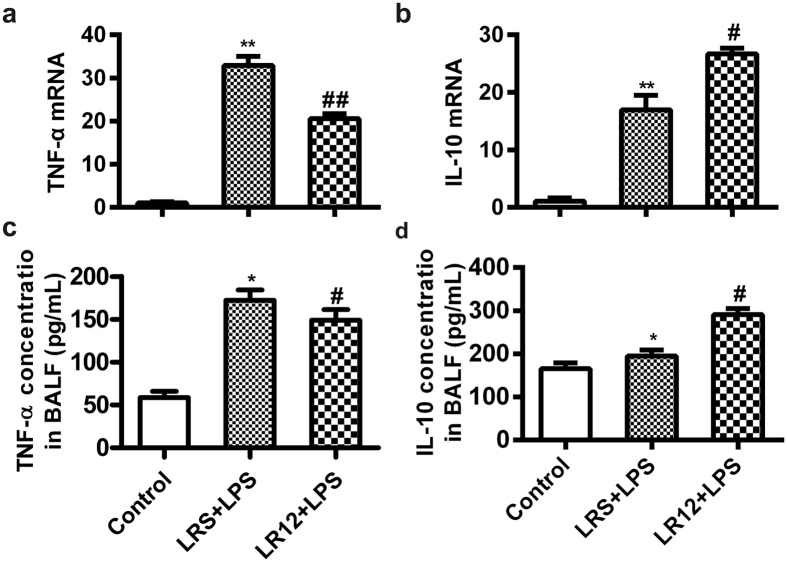
LR12 altered TNF-α and IL-10 expression in LPS-induced ALI mice. Mice received saline, LR12 (5 mg/kg) and LRS (5 mg/kg) intravenous injection, respectively. Two hours later, mice were treated with either saline or LPS (5 mg/kg, *i.t*.) for 6 h. The expression of *TNF-α* (**a**) and *IL-10* (**b**) mRNA in the lung was measured by Real-time PCR and the concentration of TNF-α (**c**) and IL-10 (**d**) in BALF were detected by ELISA. (n = 4–7). Data are expressed as the mean ± SEM. *P < 0.05 and **P < 0.01 vs Control group, ^#^*P* < 0.05 and ^##^*P* < 0.01 vs LRS+LPS group.

Overexpression of inflammatory cytokines is another important feature of ALI. Therefore, we measured the level of TNF-α and IL-10 in the lung tissue and BALF. LPS significantly up-regulated *TNF-α* mRNA level in lung tissues and protein concentration in BALF. These effects were significantly reduced by LR12 (Fig. 4a,c). LR12 also enhanced expression of anti-inflammatory cytokine *IL-10* mRNA and protein triggered by LPS injection (Fig. 4b,d).

### LR12 inhibited the expression and secretion of IL-1β in LPS-treated mice

**Figure 5:**
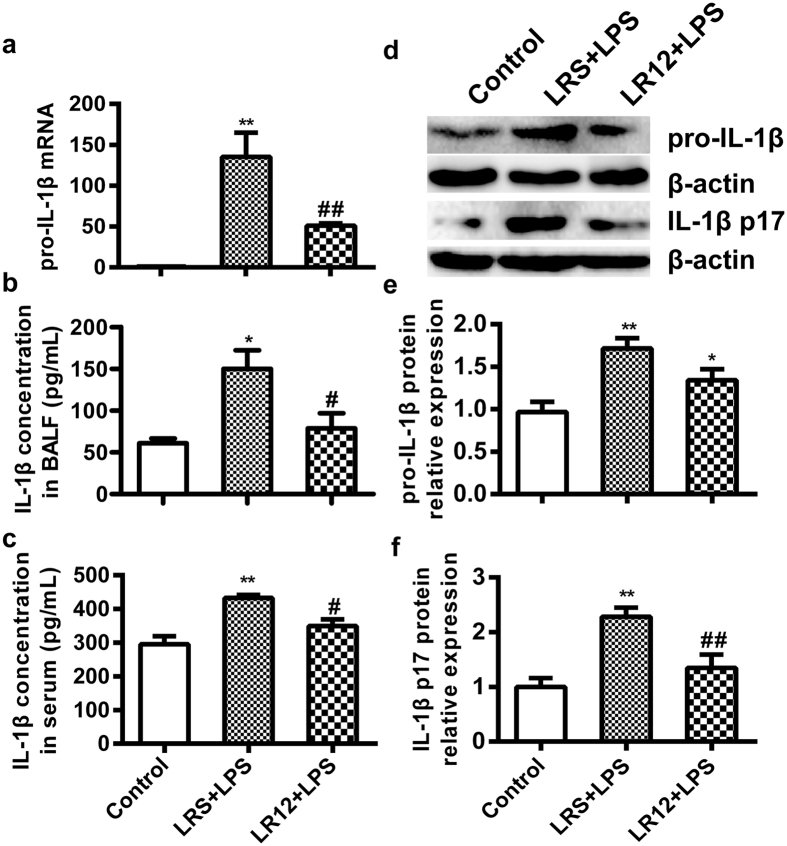
LR12 inhibited the expression and secretion of IL-1β in LPS-treated mice. Mice received saline, LR12 (5 mg/kg) and LRS (5 mg/kg) intravenous injection, respectively. Two hours later, mice were treated with either saline or LPS (5 mg/kg, *i.t*.) for 6 h. The mRNA expression of *pro-IL-1β* (**a**) was determined by Real-time PCR, and the concentration of IL-1β in BALF **(b**) and serum (**c)** were detected by ELISA. The protein level of pro-IL-1β (**d** and **e**) and IL-1β p17 (**d** and **f**) in the lung homogenate was assessed by Western blot. β-actin was used as an internal control. (n = 4–7). Data are expressed as the mean ± SEM. *P < 0.05 and **P < 0.01 vs Control group, ^#^*P* < 0.05 and ^##^*P* < 0.01 vs LRS+LPS group.

Next, we determined whether blocking the TREM-1 altered expression of inflammatory cytokine IL-1 β. The results showed that LPS increased expression of the *pro-IL-1β* mRNA and secretion of IL-1β, and LR12 inhibited remarkably LPS-induced expression of the pro-IL-1β mRNA (Fig. 5a), and lowered the level of IL-1β in BALF (Fig. 5b) and serum (Fig. 5c). Additionally, LR12 significantly reduced protein expression of pro-IL-1β and IL-1β p17, the maturation form of IL-1β, in the lung tissue of LPS-treated mice (Fig. 5d–f). As IL-1β maturation and secretion mainly depend on NLRP3 activation, these data suggest that LR12 inhibits activation of the NLRP3 inflammasome.

### LR12 suppressed NLRP3 inflammasome activation in LPS-treated mice

**Figure 6:**
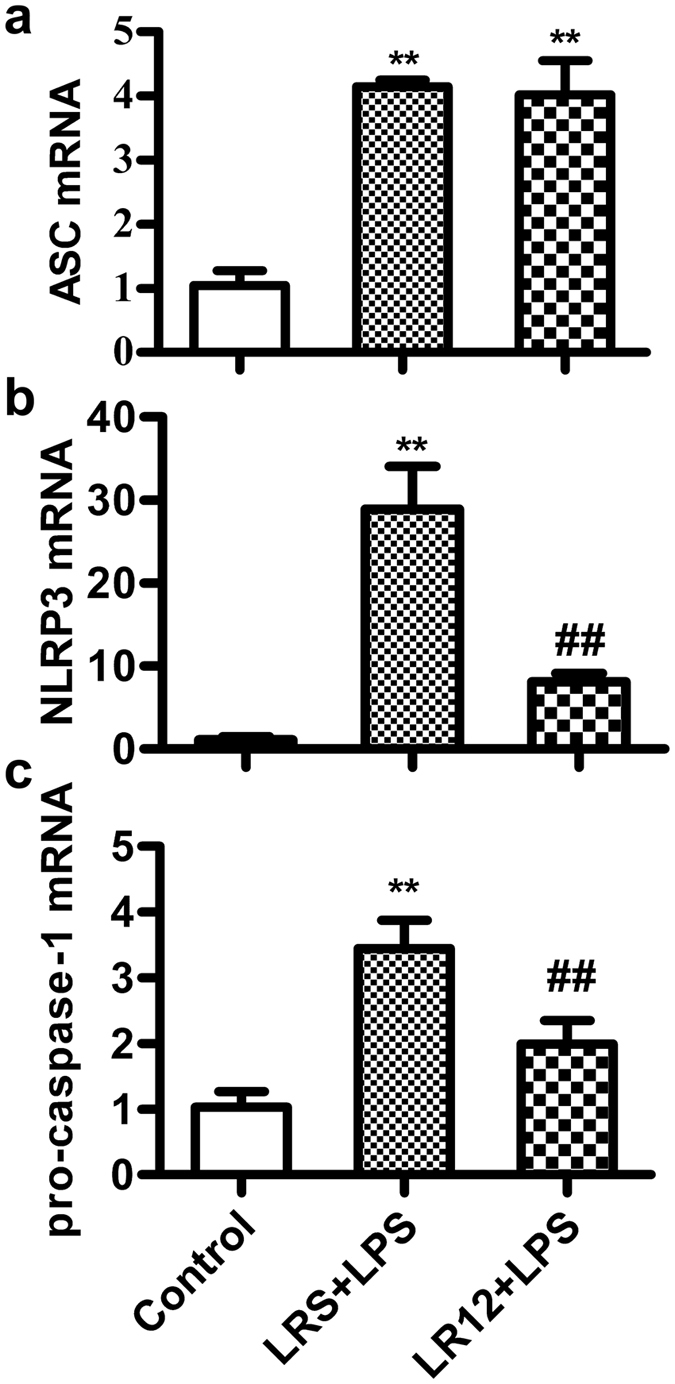
LR12 suppressed LPS-induced expression of the NLRP3 inflammasome components. Mice received saline, LR12 (5 mg/kg) and LRS (5 mg/kg) intravenous injection, respectively. Two hours later, mice were treated with either saline or LPS (5 mg/kg, *i.t*.) for 6 h. The mRNA expression of *ASC* (**a**), *NLRP3* (**b**) and *pro-caspase-1* (**c**) were determined by Real-time PCR. (n = 4–7). Data are expressed as the mean ± SEM. **P < 0.01 vs Control group, ^##^*P* < 0.01 vs LRS+LPS group.

**Figure 7:**
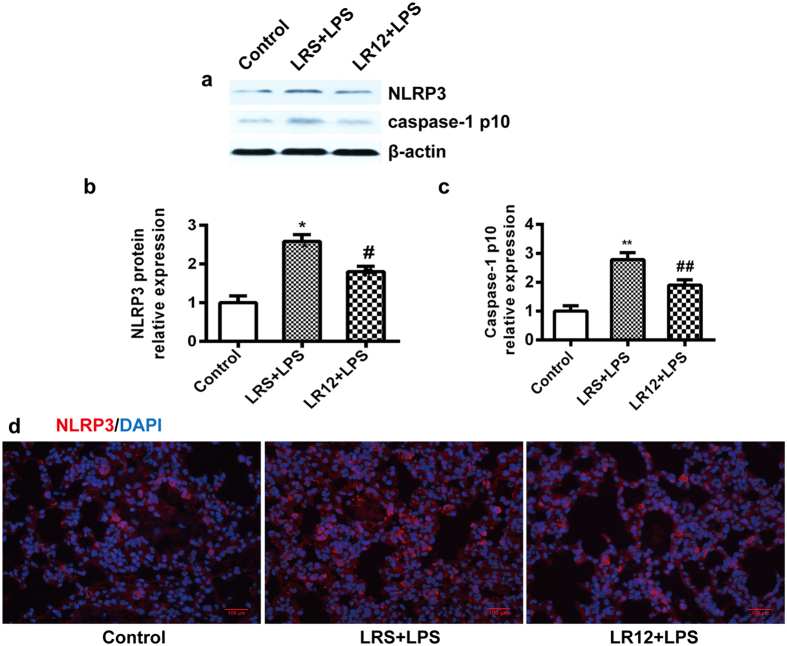
LR12 inhibited LPS-induced NLRP3 inflammasome activation. Mice received saline, LR12 (5 mg/kg) and LRS (5 mg/kg) intravenous injection, respectively. Two hours later, mice were treated with either saline or LPS (5 mg/kg, *i.t*.) for 6 h. The protein level of NLRP3 (**a** and **b**), Caspase-1 p10 (**a** and **c**) in the lung were assessed by Western-blot analysis. β-actin was used as an internal control. The immunofluorescent assay was used to detect expression of NLRP3 protein (**d**) *in situ* (n = 4–7, Bar = 100 μm). Data are expressed as the mean ± SEM. *P < 0.05 and **P < 0.01 vs Control group, ^#^*P* < 0.05 and ^##^*P* < 0.01 vs LRS+LPS group.

The NLRP3 inflammasome is a multi-protein complex comprised of NLRP3, ASC and pro-caspase-1. Expression of NLRP3 components is the priming step in NLRP3 activation^33^. In order to investigate the effect of TREM-1 on NLRP3 inflammasome priming, the expression of NLRP3, ASC and pro-caspase-1 were detected. The expression of *ASC, NLRP3* and *pro-caspase-1* mRNA (Fig. 6a,c) were increased in the lung of LPS-treated mice, and LR12 significantly lowered the expression of *NLRP3* and *pro-caspase-1* mRNA, but had no effect on expression of the *ASC* mRNA. Western-blot and immunofluorescent stains also revealed that LR12 attenuated the NLRP3 protein expression induced by LPS (Fig. 7).

Upon NRLP3 activation, pro-caspase-1 is hydrolyzed into two active fragments: caspase-1 p10 and caspase-1 p20, which cleave pro-IL-1β into IL-1β. Thus, caspase-1 p10 is a biomarker for NLRP3 activation. We found that expression of the caspase-1 p10 protein (Fig. 7a,c) was significantly increased after LPS administration. LR12 treatment markedly attenuated caspase-1 p10 expression induced by LPS.

### LR12 inhibited IκB degradation and oxidative stress in LPS-treated mice

**Figure 8:**
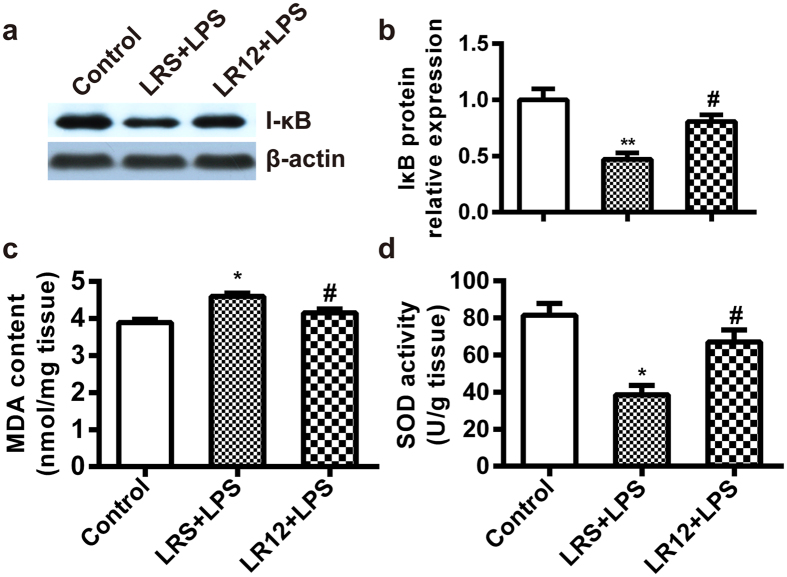
LR12 inhibited IκB degradation and oxidative stress in LPS-treated mice. Mice received saline, LR12 (5 mg/kg) and LRS (5 mg/kg) intravenous injection, respectively. Two hours later, mice were treated with either saline or LPS (5 mg/kg, *i.t*.) for 6 h. IκB protein level (**a** and **b**) in the lung was assessed by Western blot analysis. β-actin was used as an internal control. MDA (**c**) and SOD activity (**d**) were determined (n = 4–7). Data are expressed as the mean ± SEM. *P < 0.05 and **P < 0.01 vs Control group, ^#^*P* < 0.05 and ^##^*P* < 0.01 vs LRS+LPS group.

IκB degradation is an indicative of NF-κB activation, which plays a vital role in NLRP3 inflammasome priming^34^. We found that LPS significantly promoted IκB degradation, which was remarkably inhibited by LR12 (Fig. 8a,b). This data indicates that LR12 inhibits NLRP3 inflammasome priming *via* suppressing NF-κB activation.

The generation of reactive oxygen species (ROS) is one of the most important factors regulating activation of NLRP3 inflammasome. To evaluate oxidative stress, we measured MDA, a lipid peroxidation product. The MDA level in lung tissues was increased rapidly after LPS treatment. LR12 significantly decreased the amount of MDA in the lung (Fig. 8c). Furthermore, the activity of SOD, a main ROS scavenger enzyme, was examined to evaluate the antioxidant activity of LR12 in the lung tissue. SOD activity in lung tissues was decreased in LPS-treated mice, and LR12 partially restored SOD activity in these mice (Fig. 8d). These data suggest that LR12 inhibits activation of NLRP3 inflammasome *via* suppression of ROS production.

## Discussion

ALI is a life-threatening condition, which can result from bacterial infection, trauma, hemorrhagic shock, chemical inhalation, blood transfusion and aspiration pneumonia^35^. The pathological hallmark of ALI is inflammation and disruption of the lung tissues, and excessive or prolonged inflammatory processes contribute to the pathogenesis of ALI. Therefore, optimal control of the inflammatory responses appears to be an important step for the treatment of ALI^4^.

TREM-1 is an immune receptor expressed on neutrophils, macrophages and mature monocytes. It acts as an amplifier of the innate immune response and plays an important role in infectious disease^36^,^37^. Inhibition of TREM-1 activation by synthetic short inhibitory peptides or fusion protein derived from TLT-1 improves survival in various models of severe infections and myocardial injury^25^,^38^,^39^. Similar to this observation, our study showed that blocking TREM-1 with LR12 increased the survival rate and alleviated pathological changes of the lung in LPS-induced ALI mice model.

The disruption of endothelial and epithelial barriers of the lung is a hallmark of ALI, which facilitates the penetration of protein and leads to lung edema^2^. In this study, we found that LR12 reduced the disruption of endothelial-alveolar epithelial barriers and tissues damage induced by LPS, as evidenced by decreased lung wet-to-dry ratio, total protein concentration in BALF of ALI mice. The disruption of endothelial and epithelial barriers of the lung also can facilitate inflammatory cells infiltration into the alveolar space. Previous studies showed that TREM-1 can facilitate the transepithelial migration of neutrophils into the airspace, amplify the inflammatory cascade *via* enhancing the production of pro-inflammatory cytokines and chemokines in the presence of LPS^40^,^41^. Our study showed that LR12 attenuated accumulation of the inflammatory cells, expression of the pro-inflammatory cytokine TNF-α, and increased expression of the anti-inflammatory cytokine IL-10 in LPS-treated mice. These results suggest blocking TREM-1 can attenuate LPS-induced inflammation *via* improving the integrity of endothelial-epithelial barriers, inhibiting infiltration of the inflammatory cells and expression of the pro-inflammatory cytokines.

Previous study showed that TREM-1 activation can promote LPS-induced IL-1β release in human monocytes^23^. IL-1β is produced as an inactive cytoplasmic precursor after LPS stimulation that has to be cleaved to generate the mature active form (IL-1β p17). The process is highly dependent on the activation of NLRP3 inflammasomes^42^. The NLRP3 inflammasome is currently the most fully characterized inflammasome, which responds to numerous physical and chemical stimuli, and leads to a series of diseases including ALI. The NLRP3 inflammasome is comprised of the NLR protein NLRP3, the adapter ASC and pro-caspase-1. Activation of the NLRP3 inflammasome is tightly regulated at different levels. A priming signal induces synthesis of NLRP3 component and IL-1β. The level of NLRP3 protein expression has been shown to be a limiting step in controlling inflammasome activation^34^,^43^. Here, we demonstrated that LR12 inhibited expression of the NLRP3 inflammasome components mRNA and NLRP3 protein induced by LPS. These data suggested that TREM-1 activation can facilitate the NLRP3 inflammasome priming. Transcription factor NF-κB is essential for NLRP3 inflammasome priming after LPS treatment^34^. Previous studies showed that TREM-1 activation enhances TLRs responses. This augmentation requires CARD9 (the caspase-recruitment domain 9). CARD9 can form a complex with B-cell lymphoma/leukemia 10 (Bcl-10) and Mucosa-associated lymphoid tissue lymphoma translocation protein 1 (Malt1) after activation of TLRs, then activate MAPKs and lead to activation of NF-κB^44^. We postulated that NLRP3 inflammasome priming amplified by TREM-1 might be related to NF-κB activation. Our study demonstrated that LR12 can block IκB protein degradation, therefore suppress activation of NF-κB. These data suggest that TREM-1 promotes NLRP3 inflammasome priming *via* augmenting NF-κB activation in the presence of LPS.

The NLRP3 inflammasome can be activated under various conditions such as tissue injury, changes in redox homeostasis, lysosomal stability and ion concentrations etc^45^,^46^. These intracellular signals can trigger assembly of the NLRP3 inflammasome components, then hydrolyze pro-caspase-1 into two active fragments: caspase-1 p10 and caspase-1 p20^47^. Activated caspase-1 cleaves the pro-IL-1β into the active form. In this study, we found that LPS increased the expression of caspase-1 p10 and IL-1β p17 protein, and these actions were inhibited by LR12. These findings suggest that TREM-1 can promote activation of the NLRP3 inflammasome. The generation of ROS is one of the most important factors in NLRP3 inflammasome activation. Inhibition of ROS production or scavengers of ROS, such as NAD(P) H oxidase inhibitors, can suppress NLRP3 inflammasome activation^48^. ROS activates NLRP3 inflammasome in paraquat, burn, and hemorrhagic shock-induced ALI^10^,^49^,^50^. It has been reported that TREM-1 activation stimulates intracellular Ca^2+^ mobilization^11^. Excessive and/or sustained Ca^2+^ influx can trigger mitochondrial Ca^2+^ overload, leading to mitochondrial damage and high levels of mtROS production. Inhibiting Ca^2+^ mobilization can reduce mtROS production as well as activation of the NLRP3 inflammasome^51^. We hypothesized that the effect of TREM-1 on NLRP3 inflammasome activation may be associated with overproduction of ROS. Due to extremely short half-life of ROS (~15 s), we measured MDA concentration, an end product from lipid peroxidation, and SOD activity as indirect indexes for intracellular redox stress. SOD is a ROS scavenger that catalyzes O_2_^−^ into H_2_O_2_ and O_2_ to eliminate ROS^52^. In the present study, we found that LR12 reduced MDA levels and increased SOD activity after LPS treatment. These data suggest that TREM-1 may trigger NLRP3 activation *via* promoting ROS production.

In summary, we find that blocking the TREM-1 can relieve LPS-induced ALI and improve survival rate. To our knowledge, this is the first report about the regulation of NLRP3 inflammasome activation by TREM-1. This regulatory pathway might be related to activation of NF-κB and overproduction of ROS. Furthermore, TREM-1 might be a therapeutic target for the treatment of ALI.

## Additional Information

**How to cite this article**: Liu, T. *et al*. Blocking triggering receptor expressed on myeloid cells-1 attenuates lipopolysaccharide-induced acute lung injury *via* inhibiting NLRP3 inflammasome activation. *Sci. Rep.* **6**, 39473; doi: 10.1038/srep39473 (2016).

**Publisher's note:** Springer Nature remains neutral with regard to jurisdictional claims in published maps and institutional affiliations.

## Supplementary Material

Supplementary Data
