# Supplementary material for: Blocking triggering receptor expressed on myeloid cells-1 attenuates lipopolysaccharide-induced acute lung injury via inhibiting NLRP3 inflammasome activation
Source: Sci Rep. 2016 Dec 22;6:39473. doi: 10.1038/srep39473 (PMC5177963; doi:10.1038/srep39473)
Supplement: Supplementary Data [file srep39473-s1.doc]

**Blocking triggering receptor expressed on myeloid cells-1 attenuates lipopolysaccharide-induced acute lung injury *via* inhibiting NLRP3 inflammasome activation**

Tian Liu^1^, Yong Zhou^1^, Ping Li^1^, Jia-Xi Duan^1^, Yong-Ping Liu^1^, Guo-Ying Sun^1^, Li Wan^1^,

Liang Dong^1,2^, Xiang Fang^3^, Jian-Xin Jiang^4*^, Cha-Xiang Guan^1*^

^1^ Department of Physiology, Xiangya School of Medicine, Central South University, Changsha, Hunan 410078, China

^2^ Department of Anesthesiology, Affiliated Hospital of Zunyi Medical College, Zunyi, Guizhou 56300, China

^3^ Department of Neurology, University of Texas Medical Branch, Galveston, TX 77555, USA

^4^ State Key Laboratory of Trauma, Burns, and Combined Injury, Research Institute of Surgery, Daping Hospital, Third Military Medical University, Chongqing, 400042, China

**Running title:** LR12 attenuates LPS-induced ALI in mice


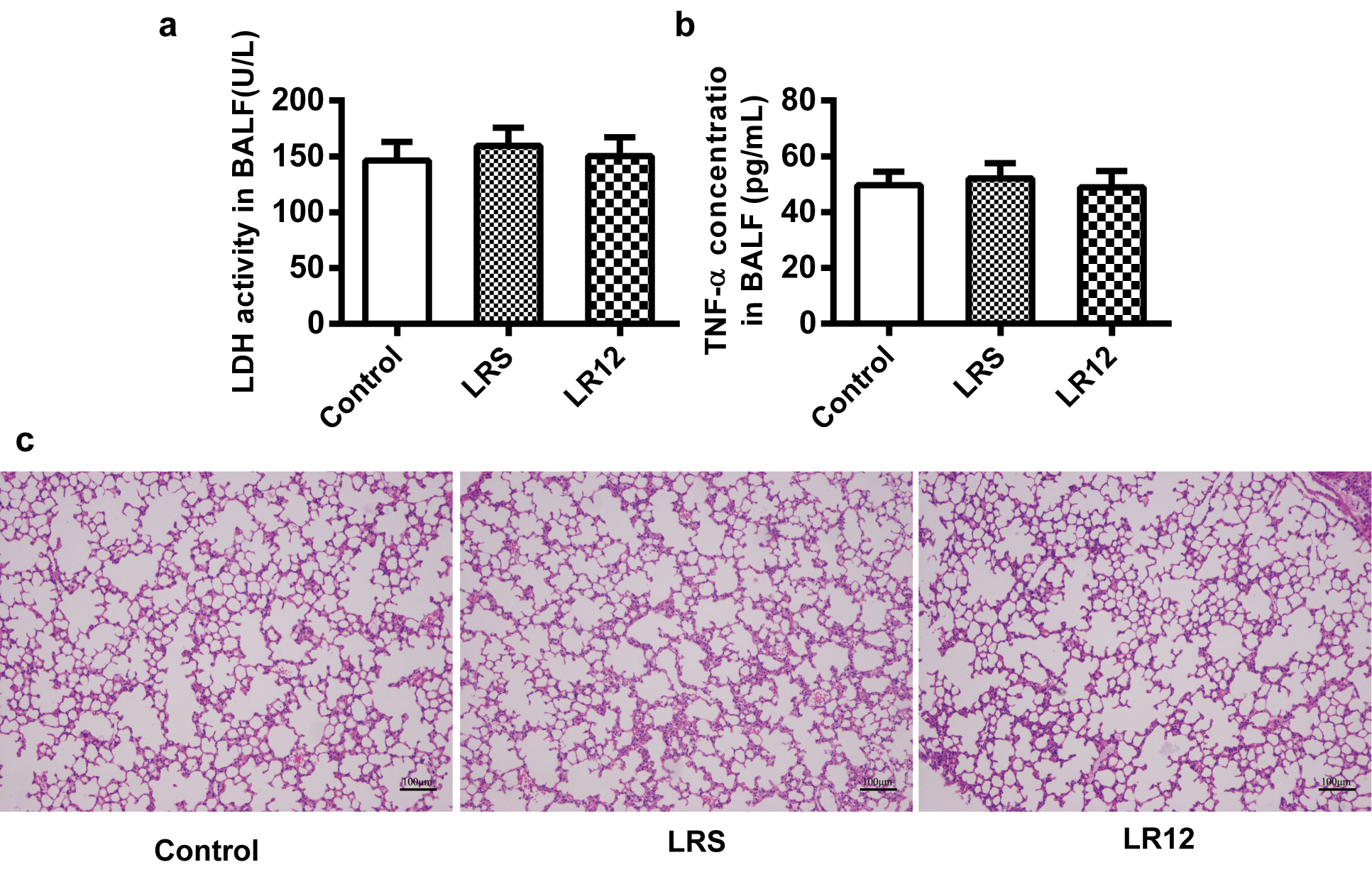


**Fig s1. LR12 and LRS have no effect on the cellular injury and lung morphology in lung of mice under normal condition.** Mice were received saline, LRS or LR12 (5 mg/kg) intravenous injection. Two hours later, mice were received saline intratracheal injection (50 μL) for 6 h. LDH activity (**a**) and TNF-α concentration (**b**) in BALF, and the morphology changes of lung (**c**) were determined. Data are expressed as the mean±SEM.
